# Studies with statistically significant effect estimates are more frequently published compared to non-significant estimates in oral health journals

**DOI:** 10.1186/s12874-022-01795-3

**Published:** 2023-01-09

**Authors:** Jadbinder Seehra, Hadil Khraishi, Nikolaos Pandis

**Affiliations:** 1 Centre for Craniofacial Development & Regeneration, Faculty of Dentistry, Oral & Craniofacial Sciences, King’s College London, Guy’s Hospital, Guy’s and St Thomas NHS Foundation Trust, Floor 25, SE1 9RT London, UK; 2 Oral & Maxillofacial Surgery and Orthodontics Clinic, Cambridge University Hospitals NHS Foundation Trust, Hills Road, Cambridge, CB2 0QQ UK; 3 Department of Orthodontics and Dentofacial Orthopedics, Dental School/Medical Faculty, University of Bern, Freiburgstrasse 7, CH-3010 Bern, Switzerland

**Keywords:** Oral health, Speciality, Significance bias, Publishing trends, Publication bias, Selective reporting

## Abstract

**Background:**

Studies reporting statistically significant effect estimates tend to be more frequently published compared to studies reporting non-significant or equivalent estimates. Consequently, this may lead to distortion of the literature. The aim of this study is to assess the prevalence of reporting statistically significant effect estimates in leading oral health journals and to explore associations between the effect estimates and record characteristics.

**Methods:**

An electronic database search was undertaken of a selection of leading oral health journals including general oral health journals to identify primary oral health records published in 2019. Descriptive statistics and population average GEE logistic regression model was used to assess associations between articles reporting a statistically significant effect estimate and the record characteristics.

**Results:**

In 1335 records, 82.4% records reported a statistically significant effect estimate. All speciality journals compared to general oral health journals were less likely to publish a record with significant effect estimates. Authors based in Asia or other (OR 1.49; 95% CI :1.02,2.19; *p* = 0.037) were more likely to report significant effect estimates compared to those based in Europe. Interventional (OR 0.35; 0.22,0.58; *p* < 0.001) and observational (OR 0.56; 0.36, 0.89; *p* = 0.013) records were less likely to report significant effect estimates compared to in-vitro studies. Registered records were less likely to report significant effect estimates when compared to non-registered studies (OR 0.22; 95% CI :0.14,0.32; *p* < 0.001).

**Conclusion:**

The publishing of records with significant effect estimates is prevalent within the oral health literature. To reduce dissemination bias and overestimation of effect sizes in systematic reviews, the publishing of studies with non-significant or equivalent effect estimates should be encouraged.

## Introduction

It is well established that studies reporting statistically significant findings are more likely to be published compared to studies reporting non-significant or equivalent findings [1, 2]. Systematic non-dissemination of studies has been reported to distort the evidence base for decision making, lead to wastage of resources and potentially have major consequences on healthcare [3]. Dissemination bias is described as when the “dissemination profile of a study’s results depends on the direction or strength of its findings” [4]. Dissemination profile is defined as the “accessibility of research results, or the possibility of research findings being identified by potential users” [4]. Publication bias which to an extent contributes to dissemination bias occurs as a result of researchers failing to write up and submit their research findings [4]. However, the decision to submit for publication can also be influenced by sponsors, journal editors’ preference and both internal and external driven factors to fulfil academic goals [4]. Ultimately, the dissemination of research findings can be viewed as a biased process. However, its impact will depend on other variables [4]. This form of bias is not uncommon with indirect evidence suggesting publication bias occurs prior to dissemination of results to the scientific community [5]. Furthermore, increasing concerns have been raised that, most research results may represent false findings and not be reflective of the “truth” [6].

It has been suggested that the desire to report significant p-values is not the main driver of publication bias within the literature but it merely reflects the incentives to report positive effects [7]. Due to competition for funding and career progression, authors may avoid publishing studies with non-significant findings. Additionally, authors may assume their findings will be deemed to be not interesting and this could influence their willingness to publish [8]. Rejection rates and bias towards studies reporting negative or non-significant results is increasing [9, 10]. Conversely, journal editors may favour accepting studies with positive results as this could increase the journal citation metrics [11]. Furthermore, the drive to publish positive findings in journals with a high Impact Factor could be seen as favourable when to both publishers and advertisers and could strengthen the application for additional funding and income [11].

Within the medical literature the trend for reporting studies with positive results does not appear to be abating with yearly increases observed [12]. Previous studies have shown the reporting of significant results in dental speciality journals to range between 71.3 and 90% [13, 14]. However, these assessments were undertaken 5–10 years ago, leaving the question if a preponderance to publish studies with positive effects is still active given the increased awareness of the problem. Therefore, the aim of this study is to assess the prevalence of reporting statistically significant effect estimates in leading oral health journals and to explore associations between the effect estimates and record characteristics.

## Methods

### Eligibility criteria

A selection of leading oral health journals (general and speciality) with the highest impact factor as published in 2019 were included in this study. No other selection criteria were used. Records published in English were included. Case reports, review articles, editorials and systematic reviews were excluded. Similar to previous investigations, studies were categorized as (1) in-vitro, (2) interventional and (3) observational [13].

### Search of oral health studies

An electronic database search was undertaken using Medline via PubMed (www.pubmed.ncbi.nlm.nih.gov) by one author (HK) in August 2020. Primary oral health records (in-vitro and in-vivo) published between 1st January 2019 and 31st December 2019 in the following journals were sourced: Journal of Dental research (JDR), Journal of American Dental Association (JADA), European Journal of Orthodontics (EJO), American Journal of Orthodontics and Dentofacial Orthopaedics (AJODO), Journal of Clinical Periodontology (JCP), Journal of Periodontology(JOP), Journal of Endodontics (JOE), International Journal of Oral and maxillofacial surgery (IJOMS), Journal of Oral and Maxillofacial Surgery (JOMS), Pediatric dentistry (PD), European Journal of Paediatric dentistry (EJPD), Journal of Prosthetic Dentistry (JPD) and Journal of Prosthodontics (JOPR). Field tags were not employed. The full record titles were searched without any language filters. The date limit function was used to identify records published within the study timeframe. The titles and abstracts of records (case reports, reviews, editorials, and systematic reviews) not meeting eligibility criteria were excluded during the screening process. All titles and abstracts were screened independently by 2 authors (HK and JS). Full-text records of abstracts fulfilling the inclusion criteria were retrieved and further analysed for eligibility independently by two authors (HK and JS). Any disagreements in the final records were resolved by discussion among the authors.

### Data extraction

A pilot assessment of 10 random records was undertaken between two authors (HK and JS) to ensure consistency in data extraction variables. All record characteristics were extracted by a single author (HK) and entered into a pre-piloted Microsoft Excel® (Microsoft, Redmond, WA) data collection sheet. A second author (JS) independently cross-checked the collected data. Any discrepancies were resolved by discussion. At the level of each record the following characteristics was extracted: journal title, continent of corresponding author, journal impact factor, speciality of journal, study type (in-vitro, interventional, observational), ethical approval (approval obtained or not reported). When the relevant information was not reported it was assumed that no approval was required, or the project was exempt from approval), involvement of statistician (yes or no; inferred from author affiliations and materials and methods section), significance of effect estimate (based on primary outcome. In the absence of no clear primary outcome, the first outcome was analysed: significant or non-significant.), study registration (yes or no) and conflict of interest (yes conflict of interest is present/declared, or no conflict of interest is present/declared).

### Statistical analysis

Descriptive statistics on the characteristics of the records were calculated. A population average univariable GEE logistic regression model with an exchangeable correlation structure was fit to assess associations between records reporting a statistically significant effect estimate and the record characteristics (independent variables). Estimates, corresponding 95% CIs and p-values were calculated. Significant predictors identified during the univariable analysis were entered in the multivariable model. The variable journal was used as the clustering unit. Statistical significance was set at 0.05 (2-sided). All statistical analyses were performed using STATA software version 16.1 (Stata Corporation, College Station, Texas, USA) and R Software version 3.6.1 (R Foundation for Statistical Computing, Vienna, Austria).

## Results

**Fig. 1:**
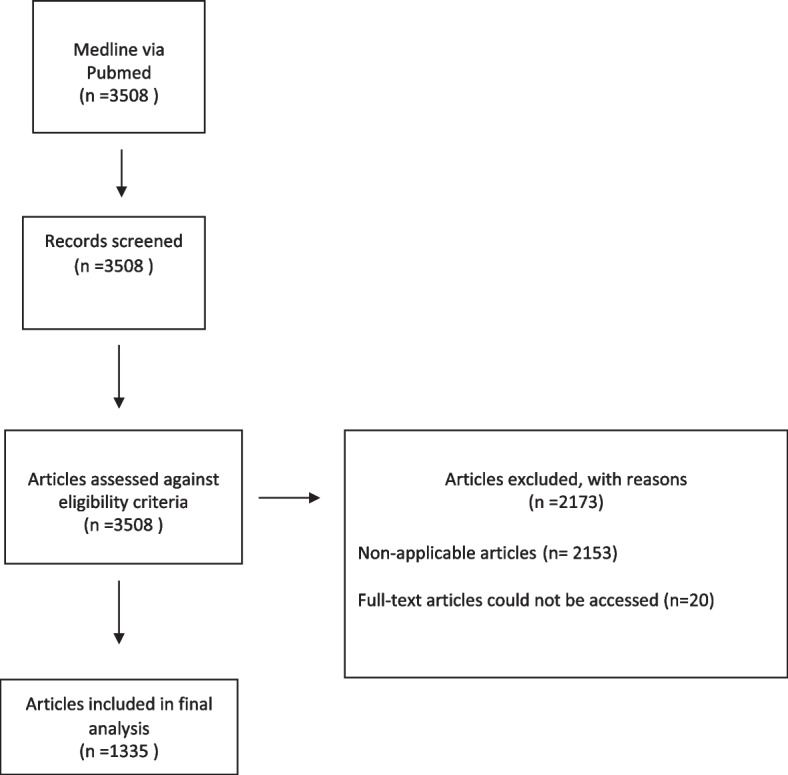
Study identification flow diagram

A total of 1335 records were included in this study (Fig. 1).

Over 82% (n = 1101) of the records reported a statistically significant effect estimate. Within this cohort, the highest number of records reporting a significant effect estimate were published in JDR (97.8%) with the lowest number published in EJO (67.9%). The range of reporting significant effect estimates (highest-lowest) for the following variables were: specialty journal (general oral health journals (96.8%)-orthodontics (73.7%), continent of corresponding author (Asia or other (85.3%)-Europe (76.5%) and study type (in-vitro (90.8%) - interventional (67.1%). In records reporting significant effect estimates, ethical approval was not reported (87.0%), did not involve a statistician (82.8%), were not registered (85.2%) and reported a conflict of interest (84.0%) (Table 1).

**Table 1:** Record characteristics reported by significance of effect estimate.: non-significant (n = 234) and significant (n = 1101). The distribution of articles by specialty of journal, study type (in-vitro, interventional, observational) and significance of results are shown in Fig. 2

**Variable**	**Non-significant** ***N*** **(%)**	**Significant*N*** **(%)**
Journal title		
JDR	2 (2.2)	90 (97.8)
JADA	2 (6.1)	31 (93.9)
EJO	18 (32.1)	38 (67.9)
AJODO	22 (22.9)	74 (77.1)
JCP	26 (27.7)	68 (72.3)
JOP	24 (13.3)	156 (86.7)
JOE	18 (13.3)	117 (86.7)
IJOMS	40 (28.2)	102 (71.8)
JOMS	38 (15.8)	203 (84.2)
PD	5 (25.0)	15 (75.0)
EJPD	4 (19.0)	17 (81.0)
JPD	26 (15.3)	144 (84.7)
JOPR	9 (16.4)	46 (83.6)
Specialty Journal		
General oral health journals	4 (3.2)	121 (96.8)
Orthodontics	40 (26.3)	112 (73.7)
Periodontology	50 (18.2)	224 (81.8)
Endodontics	18 (13.3)	117 (86.7)
Oral and Maxillofacial surgey	78 (20.4)	305 (79.6)
Pedatrics	9 (22.0)	32 (78.0)
Prosthodontics	35 (15.6)	190 (84.4)
Continent of corresponding author		
Europe	72 (23.5)	235 (76.5)
Americas	80 (17.1)	389 (82.9)
Asia or other	82 (14.7)	477 (85.3)
Study type		
In-vitro	42 (9.2)	417 (90.8)
Interventional	91 (32.9)	186 (67.1)
Observational	101 (16.9)	498 (83.1)
Ethical approval		
Not reported	43 (13.0)	287 (87.0)
Approval obtained	191 (19.0)	814 (81.0)
Involvement of statistician		
No	212 (17.2)	1020 (82.8)
Yes	22 (21.4)	81 (78.6)
Study registration		
No	180 (14.8)	1034 (85.2)
Yes	54 (44.6)	67 (55.4)
Conflict of interest		
No	226 (17.6)	1059 (82.4)
Yes	8 (16.0)	42 (84.0)

**Fig. 2:**
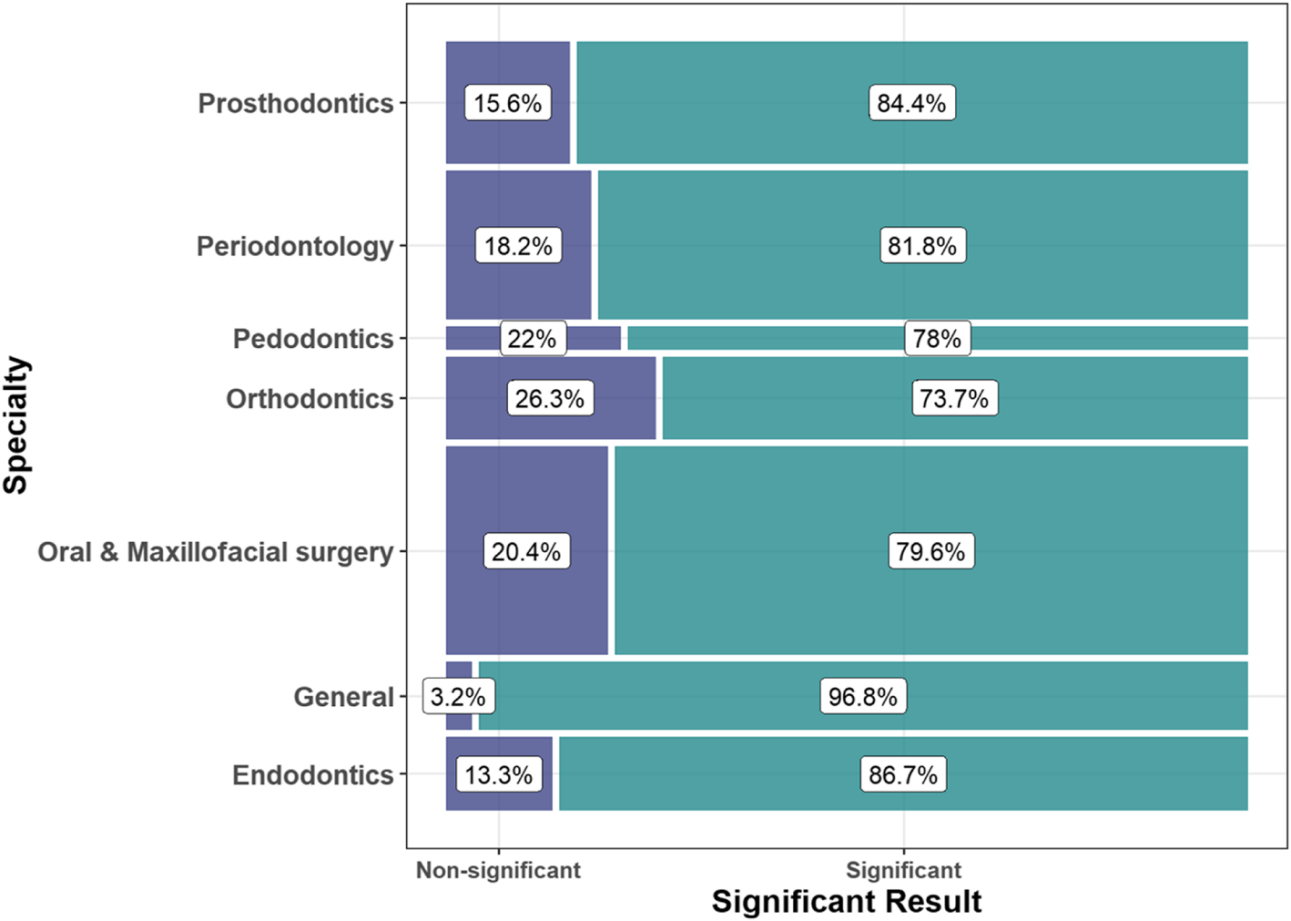
Distribution of articles by specialty of journal, and significance of effect estimate

In the multivariate analysis, all speciality records compared to general oral health types were less likely to publish a study with significant effect estimates. The continent of the corresponding author was associated with the odds of publishing an article with significant effect estimates, with authors based in Asia or other (OR 1.49; 95% CI :1.02,2.19; *p* = 0.037) being more likely to publish significant effect estimates compared to those based in Europe. In addition, interventional (OR 0.35; 0.22,0.58; *p* < 0.001) and observational (OR 0.56; 0.36, 0.89; *p* = 0.013) studies were less likely to report significant effect estimates compared to in-vitro studies. Finally, studies that were registered were less likely to report significant effect estimates when compared to non-registered studies (OR 0.22; 95% CI :0.14,0.32; *p* < 0.001) (Table 2).

**Table 2:** Univariable and multivariable GEE logistic regression derived Odds Ratios (OR) and 95% confidence intervals (CI) for the effect of speciality journal, continent of corresponding author, study type, ethical approval, involvement of statistician, study registration, conflict of interest and impact factor on the likelihood of reporting a significant result

**Predictor variables**	Category	Univariate analysis		Multivariate analysis	
		OR (95%CI)	*p*-value	OR (95%CI)	*p*-value
Speciality journal	General oral health journals	Reference			
	Orthodontics	0.09 (0.03,0.36)	0.001	0.13 (0.04, 0.46)	0.001
	Periodontology	0.15 (0.04, 0.55)	0.004	0.23 (0.07, 0.78)	0.018
	Endodontics	0.24 (0.06, 0.98)	0.05	0.19 (0.05, 0.73)	0.015
	Oral and Maxillofacial surgery	0.43 (0.04, 0.48)	0.002	0.15 (0.04, 0.49)	0.002
	Pedatrics	0.13 (0.03,0.56)	0.006	0.15 (0.04, 0.60)	0.008
	Prosthodontics	0.19 (0.05, 0.74)	0.016	0.14 (0.04, 0.49)	0.002
Continent of corresponding author	Europe	Reference			
	Americas	1.26 (0.87, 1.81)	0.215	1.05 (0.72, 1.54)	0.793
	Asia or other	1.60 (1.12, 2.30)	0.01	1.49 (1.02,2.19)	0.037
Study type	In-vitro	Reference		Reference	
	Interventional	0.22 (0.15, 0.34)	<0.001	0.35 (0.22,0.58)	<0.001
	Observational	0.53 (0.35, 0.80)	0.003	0.56 (0.36, 0.89)	0.013
Ethical approval	Not reported	Reference			
	Approval obtained	0.69 (0.48, 1.02)	0.06		
Involvement of statistician	No	Reference			
	Yes	0.73 (0.45, 1.19)	0.206		
Study registration	No	Reference		Reference	
	Yes	0.22(0.14, 0.32)	<0.001	0.32 (0.19,0.53)	<0.001
Conflict of interest	No	Reference			
	Yes	1.05 (0.49, 2.21)	0.897		
Impact factor	Per unit	1.32 (0.96,1.81)	0.08		

## Discussion

This study assessed the prevalence of reporting statistically significant effect estimates in leading oral health journals and examined associations between the direction of the results and record characteristics. In the study timeframe, 82.4% full-text records reported a statistically significant effect estimate confirming the apparent preponderance towards publishing positive results within the oral health literature. The odds of publishing significant effect estimate were also associated with the continent of the corresponding author, the record type and registration of the record. This result is comparable to previous investigations which reported the reporting of significant results in dental speciality journals to range between 47 and 86% [13] and 75–90% [14]. However, the same trend is not evident in the publishing of dental abstracts where the significance of the results does not predict the likelihood of publication [15]. Although, the current study was conducted after a significant time lag between previous investigation [13, 14], it appears the publishing of records with significant effect estimates still dominates and may have increased over this time period. Indeed, investigations within medical speciality journals have found a highly significant trend of reporting positive results increasing on a yearly basis by 6% [12].

There was variation in the percentage reporting of significant effect estimates between the leading oral health journals included in this study. In addition, all speciality journals compared to general oral health types were less likely to publish positive results. As previously postulated, this could be a reflection of differences in the study types published in each journal [13]. Articles published by authors based in Asia or other were more likely to publish records with positive findings compared to those based in Europe which mirrors the findings of previous studies [12, 13, 16]. This maybe reflective of the fact that trials carried out in developing countries are reported to show more positive findings compared to trials performed in developed countries [17]. Registration of trials is encouraged to improve transparency in the conduct of the study but also to eliminate publication and selective reporting bias [18, 19]. Interestingly, registration of records was less likely to be associated with the reporting of significant effect estimates when compared to non-registered records which may suggest that registration is having the desired impact. This is also supported by the finding that at the study level, interventional type studies which are encouraged to be registered are less likely of reporting positive findings compared to in-vitro studies. This corroborates the findings of a similar studies [13]. This really highlights the importance of correct interpretation of studies with a perceived weaker design as they are more likely to report exaggerated treatment effects whereas interventional studies such as Randomised Clinical Trials can contradict the findings reported by observational studies [20, 21].

The reproducibility of research study design is reported to be poor [22]. Conversely, if replication of study design can be achieved, the results of such studies are more likely to contradict the reported initial stronger results over time, independent of the study design [23]. If an improvement in research methodology is excluded, then reporting of a significant result could be dependent on other factors [24]. First of all, it could be the fact that hypotheses tested are true. However, this needs to be balanced with the fact that authors may be confirming known hypothesis in order to get “publishable’ results [6]. Authors who detect non-significant results decline to submit for publication or these results are turned into a positive direction through post-hoc analyses, selective reporting and reinterpretation [12]. The consequences of selective reporting or “p-hacking” where investigators carry out multiple statistical tests and then report only those which produce significant results has been highlighted [7].

Although every attempt was made to elicit the primary outcome from each record, when it was not obvious, the first outcome was analysed which introduces a degree of subjectivity and potential interpretation of misleading outcome results. The presence SPIN, where beneficial effects of an intervention are highlighted despite a non-significant difference detected between treatment interventions has been established in dental speciality trials [25–27]. Indeed, SPIN related to the focusing on significant within-group comparisons, focusing on a significant primary outcome when there are several co-primary outcomes and focusing on significant secondary outcomes has been reported in orthodontic trial abstracts [25]. To avoid any subjectivity, future assessments could review the registration record, published protocol, or duplicate publication, when the primary outcome is not specifically reported.

The selected in-vitro records also include animal studies and the only articles excluded were case reports, review articles, editorials and systematic reviews. The aim of our study was to assess the prevalence of reporting significance results in oral health journals and to see if this known problem still persists. The justification for the inclusion of in-vitro records is that they include an experiment, record an outcome and commonly include the results of statistical tests. Statistically significant effect estimates in in-vitro studies can influence decisions in conducting other similar or higher-level studies. In the analysis, it is also interesting to see that statistically significant records are more prevalent in in-vitro records compared to clinical studies. For example, there is evidence that RCTs which are usually the most rigorous studies have the lowest prevalence of significant results which may imply that in vitro studies may pass under the radar and thus more attention should be paid when interpreting the in vitro studies results apart from whether the results are generalizable due to the in-vitro setting.

Reasons for non-publication of records despite the strength of the findings include lack of time, incomplete study status, low priority and issues with co-authors [15]. However, researcher related factors are primarily cited [15, 28]. In the investigation of publication of abstracts following presentation at a biomedical conference, the most frequently cited reason by authors to not publish was a lack of time [29]. Methods to encourage publishing of non-significant findings could be suggested at the study ethical approval stage. For instance, ethics committees could suggest the reporting of the results of clinical studies regardless of the direction of the effect [30]. Mandatory registration of clinical trials, enforcing guidelines for accurate reporting and creating journals of negative results have also been suggested [4, 31–34]. Regarding the latter, such journals could be funded by public or charitable support [11]. Furthermore, trial funding agencies could make it a pre-requisite to publish both significant and non-significant findings of primary hypotheses tested [11]. The disclosure of funders or reporting of funding sources was not collected as a variable in this study. Future studies, should consider this characteristic and its relationship on the publication of studies with positive results, as trials with a high or unclear risk of sponsorship bias are reported to be associated with larger treatment effect size estimates [35].

## Limitations

Searching of relevant records was conducted electronically rather than hand searching of journal issues. This decision was influenced by limitations regarding the access of hand copy journals within library institutions. Furthermore, records were only sourced from high impact oral health journals. Both these factors may have resulted in potential non-identification of potentially relevant records and selection bias. Although, the study timeframe was limited to twelve months only, we feel the total number of records included in this study represents a large enough sample to allow us to gauge the current issue of publication bias within the oral health literature [14]. Data extraction of the whole sample was primarily undertaken by a single author, but an initial calibration and cross-check for any discrepancies of the collected data by a second author has reduced errors in reporting and classification.

## Conclusion

The publishing of records with significant effect estimates is prevalent within the oral health literature. To reduce dissemination bias and overestimation of effect sizes in systematic reviews, the publishing of records with non-significant or equivalent effect estimates should be encouraged. Methods to facilitate this include ethics committees or funding agencies insisting on the reporting of the results of records regardless of the direction of the effect, mandatory registration of clinical trials, enforcing guidelines for accurate reporting and creating journals with the remit of publishing non-significant effect estimates.

## Data Availability

The datasets used and analyzed during the current study are available from the corresponding author on reasonable request.
